# Supplementary material for: GlypNirO: An automated workflow for quantitative N- and O-linked glycoproteomic data analysis
Source: Beilstein J Org Chem. 2020 Sep 1;16:2127–35. doi: 10.3762/bjoc.16.180 (PMC7476601; doi:10.3762/bjoc.16.180)
Supplement: File 2 — GlypNirO workflow overview. [file Beilstein_J_Org_Chem-16-2127-s002.pdf]

BEILSTEIN JOURNAL OF ORGANIC CHEMISTRY

Supporting Information

for

GlypNirO: An automated workflow for quantitative *N*- and *O*-linked glycoproteomic data analysis

Toan K. Phung, Cassandra L. Pegg and Benjamin L. Schulz

Beilstein J. Org. Chem. **2020,** *16,* 2127–2135. doi:10.3762/bjoc.16.180

GlypNirO workflow overview

License and Terms: This is a supporting information file under the terms of the Creative Commons Attribution License (https://creativecommons.org/licenses/by/4.0). Please note that the reuse, redistribution and reproduction in particular requires that the authors and source are credited.
The license is subject to the *Beilstein Journal of Organic Chemistry* terms and conditions: (https://www.beilstein-journals.org/bjoc)

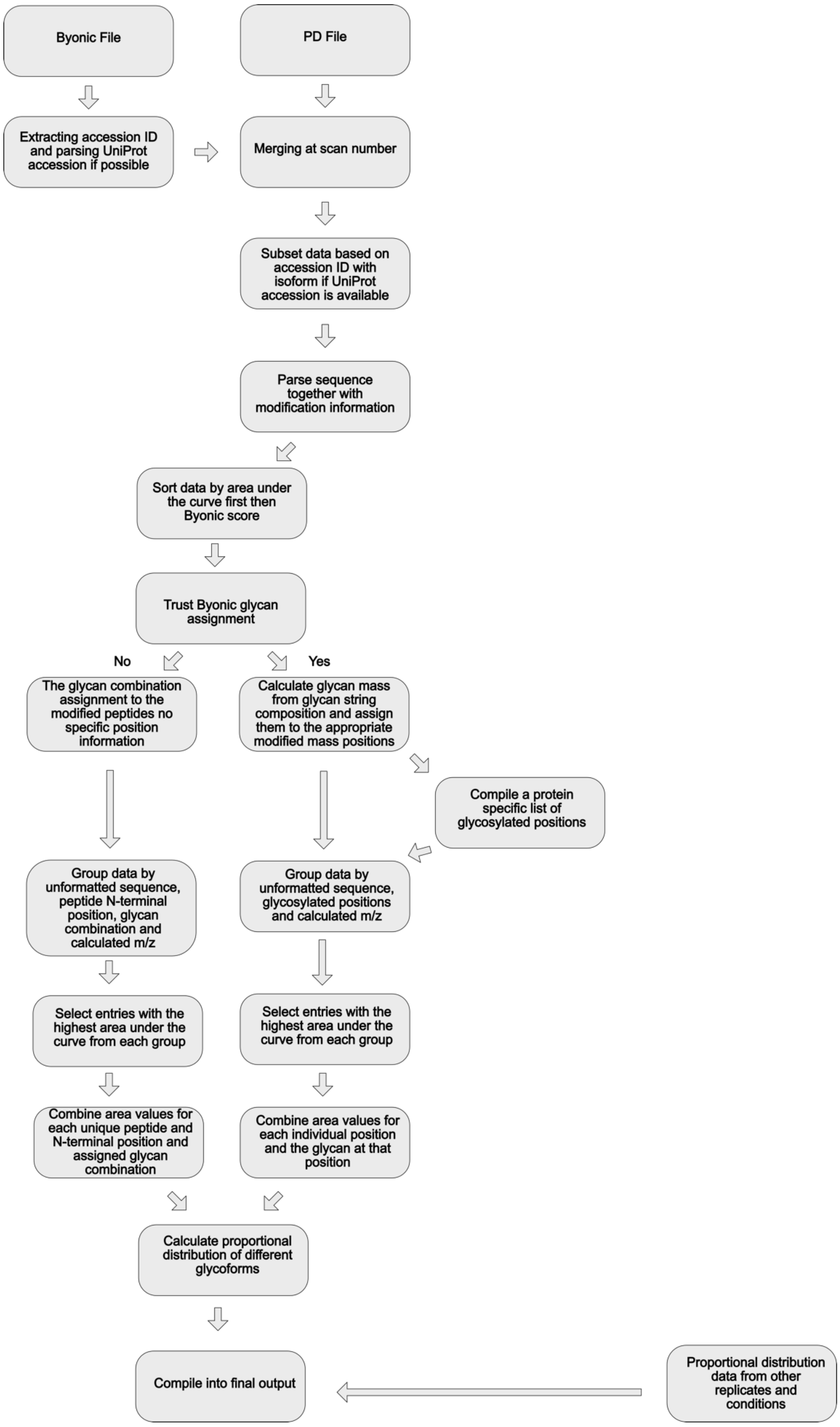

Supplementary Figure S1: GlypNirO workflow overview.
